# Sestrins at the Interface of ROS Control and Autophagy Regulation in Health and Disease

**DOI:** 10.1155/2019/1283075

**Published:** 2019-05-07

**Authors:** Marco Cordani, Miguel Sánchez-Álvarez, Raffaele Strippoli, Alexandr V. Bazhin, Massimo Donadelli

**Affiliations:** ^1^Instituto Madrileño de Estudios Avanzados en Nanociencia (IMDEA Nanociencia), CNB-CSIC-IMDEA Nanociencia Associated Unit “Unidad de Nanobiotecnología”, Madrid 28049, Spain; ^2^Mechanoadaptation & Caveolae Biology Lab, Cell and Developmental Biology Area, Centro Nacional de Investigaciones Cardiovasculares (CNIC), Madrid 28029, Spain; ^3^Department of Cellular Biotechnologies and Hematology, Section of Molecular Genetics, Sapienza University of Rome, Rome, Italy; ^4^Gene Expression Laboratory, National Institute for Infectious Diseases “Lazzaro Spallanzani” I.R.C.C.S., Rome, Italy; ^5^Department of General, Visceral and Transplantation Surgery, Ludwig Maximilian University, Munich, Germany; ^6^Department of Neurosciences, Biomedicine and Movement Sciences, Section of Biochemistry, University of Verona, Verona, Italy

## Abstract

Reactive oxygen species (ROS) and autophagy are two highly complex and interrelated components of cell physiopathology, but our understanding of their integration and their contribution to cell homeostasis and disease is still limited. Sestrins (SESNs) belong to a family of highly conserved stress-inducible proteins that orchestrate antioxidant and autophagy-regulating functions protecting cells from various noxious stimuli, including DNA damage, oxidative stress, hypoxia, and metabolic stress. They are also relevant modulators of metabolism as positive regulators of the key energy sensor AMP-dependent protein kinase (AMPK) and inhibitors of mammalian target of rapamycin complex 1 (mTORC1). Since perturbations in these pathways are central to multiple disorders, SESNs might constitute potential novel therapeutic targets of broad interest. In this review, we discuss the current understanding of regulatory and effector networks of SESNs, highlighting their significance as potential biomarkers and therapeutic targets for different diseases, such as aging-related diseases, metabolic disorders, neurodegenerative diseases, and cancer.

## 1. Introduction

Reactive oxygen species (ROS) can play essential roles as intra- and extracellular messengers, encoding the functional/metabolic state of the cell for the regulation of numerous signalling pathways. However, ROS are also powerful oxidizing agents, which can induce cell injury upon modification of lipids, proteins, or DNA, disrupting cell function and increasing the risk of DNA mutation and tumorigenesis. Oxidation of specific amino acid residues in different metabolic enzyme systems (such as the 2-oxoglutarate dehydrogenase complex in the tricarboxylic acid cycle) can alter their activity by orders of magnitude, completely changing cell sensitivity to other environmental conditions, such as fuel availability or usage of nutrients [1]. Thus, aberrant ROS levels are a consequence shared by a broad list of pathologies, and ROS dysregulation substantially drives the onset and progression of a number of diseases. For example, high ROS levels found in most cancer cells can promote metabolic rewiring and growth dysregulation, as well as aberrant response of cells to different challenges by gating the activation threshold of apoptosis, necrosis, or autophagic death. Analogies can thereby be drawn for aging biology. As such, intervention of ROS levels has received substantial attention as a potential antiaging and anticancer therapeutic opportunity, including it in the renewed study of strategies, such as differential ascorbate toxicity [2–4]. Conversely, ROS deficiency has been associated mechanistically with immune disorders, inflammation, and decreased proliferative response, partly because of the disruption of cell signalling wiring [5].

A major theme in ROS-associated disorders is their interplay with systems determining energy and nutrient homeostasis in the cell. The mechanistic target of rapamycin complex 1 (mTORC1) and 5′ AMP-activated protein kinase (AMPK) interpret multiple cues, including oxidative stress, to integrate them with the control of energy management, anabolism, and cell growth. Conversely, these signalling systems regulate metabolism and growth, which are major ROS sources themselves. These pathways, together with other stress signalling routes such as the Unfolded Protein Response (UPR), tightly regulate the autophagy flux, a key node for both the regulation of ROS levels and ROS-dependent cell regulation. This recycling function curbs ROS overproduction and, through a number of input pathways, is itself sensitive to existing ROS levels in the cell.

However, our understanding of the interplay between these two aspects of cell physiology (ROS and autophagy) is still limited. In this review, we aim to provide an overview of our current knowledge on sestrins (SESNs), a family of stress surveillance proteins which may hold a key to the integration of ROS control and autophagy regulation and may constitute an interesting source of novel therapeutic opportunities.

## 2. The Sestrin Protein Family

SESNs are a family of proteins induced upon various stressing conditions, such as hypoxia and metabolic imbalances [6]. Only one member is present in invertebrates (such as *Caenorhabditis elegans* (cSESN) and *Drosophila melanogaster* (dSESN), whereas three members are present in mammals, such as SESN1, SESN2, and SESN3. Vertebrate SESN1 (also known as PA26) is a transcriptional target of p53 [7]. SESN2 (also known as H195) was discovered as a gene activated by hypoxia [8]. The SESN3 gene is a largely uncharacterized open reading frame identified by homology [8]. Curiously, SESNs were named SESNs after a human genetics course held in Sestri Levante, a small town on the Ligurian coast of Italy, where researchers discovered the amino acid sequence homology between the three proteins [9]. Intriguingly, although they have close homology and likely common origin, each SESN gene maps to a different chromosome in the human genome: SESN1 to 6q21, SESN2 to 1p35.3, and SESN3 to 11q21 [8].

### 2.1. Structure-Function Relationships and Interactomes of SESNs

While phenotypic and pathophysiological associations for SESNs rapidly accumulated, information about their molecular underpinnings has been scarce. Inference from *in silico* studies has been limited by the fact that these proteins do not contain obvious similarity with any known structural domain or catalytic motif [6].

Recently, the determination of the human SESN2 structure by X-ray crystallography brought a novel insight into its potential function. The crystal structure revealed that hSESN2 contains two structurally similar subdomains, SESN-A and SESN-C, connected by a helix-loop-helix domain (SESN-B). Both subdomains share significant homology with proteins belonging to the alkyl hydroperoxidase family (including, for example, *M. tuberculosis* AhpD), which catalyse the reduction of peroxiredoxins [10, 11]. Alkyl hydroperoxidase activity has been confirmed for SESN2 (as reported below), while no biochemical characterization has so far been gathered for SESN1 and SESN3.

Multiple protein-protein interactions mediate activation, modulation, and function of SESNs [12]. In particular, analysis of the SESN2 structure allows the identification of three functional domains [10, 11]. An oxidoreductase active site within SESN-A, which contains the catalytic cysteine (C125) and conserved residues of the proton relay system (Y127 and H132), has been identified. These three amino acid residues were found to be critical for the antioxidant function of hSESN2. Although structural and biochemical evidence demonstrates that hSESN2 has intrinsic peroxidase activity, the physiological ROS substrate of hSESN2 remains to be identified. Secondly, a GATOR2-interacting surface at the C-terminal domain, containing a characteristic aspartate-aspartate (DD) motif, has been mapped. This DD motif appears to be required for the interaction between hSESN2 and GATOR2. The heteropentameric GATOR2 complex is a direct physical target of SESNs and mediates its mTORC1-regulating functions.

Finally, a leucine-binding site is present in the SESN-C domain. The existence of this leucine-binding site suggests that hSESN2 acts as a direct cellular sensor of leucine level, which is a relevant feature considering that leucine is a key amino acid for the lysosome-mTORC1 system to sense overall levels of amino acid nutrients [13].

**Figure 1:**
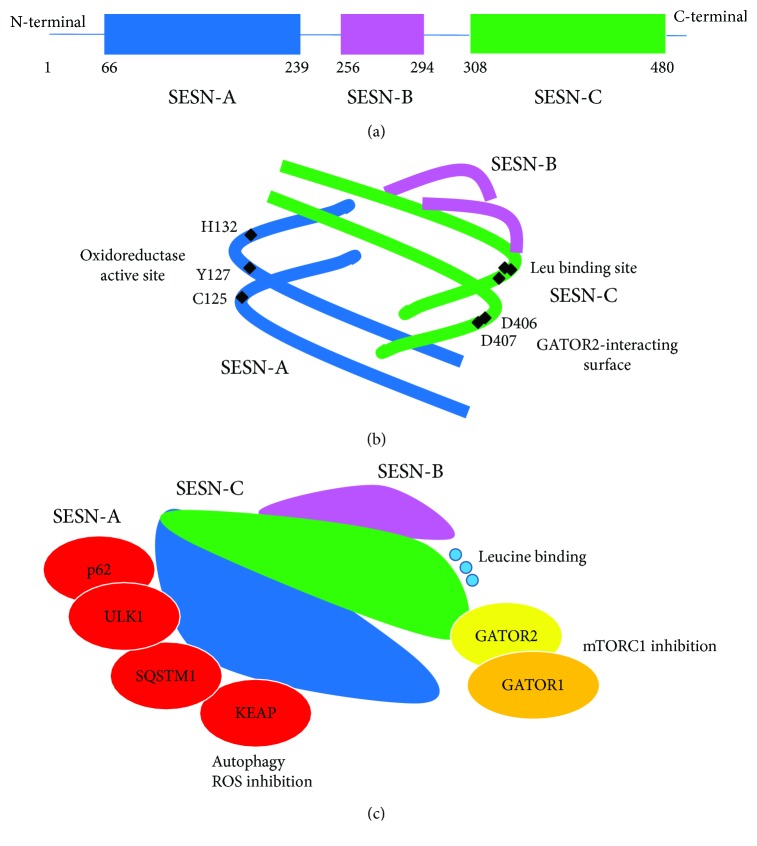
(a) Schematic diagram of full-length SESN2 showing the three domains SESN-A, SESN-B, and SESN-C. (b) Schematic representation of SESN2 showing the localization of the catalytic cysteine (C125) and conserved residues of the proton relay system (Y127 and H132) in the SESN-A domain, the characteristic aspartate-aspartate (DD) motif, and the Leu binding site in the SESN-C domain (based on structure analysis published by Ho et al. in reference [12], Figure 1, doi:10.1016/j.tibs.2016.04.005). (c) Schematic representation of SESN2 showing direct interactors and pathways involved.

Importantly, SESN2 also interacts with Kelch-like ECH-associated protein 1 (Keap1) and the autophagy regulators p62/sequestosome-1 (SQSTM1) and Unc51-like 1 (ULK1). These interactions likely underpin a substantial share of the impact of SESN2 on the suppression of oxidative damage and on autophagy regulation respectively, as detailed below (Figure 1).

### 2.2. Regulation of Expression and Function of SESNs

SESNs are proteins ubiquitously expressed in adults, although there exist tissue-specific variations in gene expression levels [7–9]. Interestingly, SESN1 and SESN2 are most highly expressed in skeletal muscle [7], a feature shared only by the *Drosophila* homolog, dSESN [14]. dSESN expression increases with aging [14], while expression of SESNs in humans is reduced in individuals of advanced age [15].

As a family of stress-inducible proteins, SESNs have been reported to be upregulated and activated upon exposure to DNA damage, oxidative stress, and hypoxia. Whereas SESN1 and SESN2 are mainly responsive to p53 [7, 8], SESN3 is activated by forkhead box O (FoxO) transcriptional factors [16]. Other transcription factors responsible for the expression of SESNs include nuclear factor erythroid 2-like 2 (Nrf2) [17], NH(2)-terminal kinase (JNK)/c-Jun pathway [18], and hypoxia-inducible factor-1*α* (HIF-1*α*) [19].

Different stimuli modulate the SESN phosphorylation state, a key aspect of SESN regulation. Multiple phosphor-acceptor sites have been found in dSESN *in vivo* in *Drosophila* [20]. In humans, regulation by phosphorylation has been characterized mainly for the SESN2 paralog. Mass spectrometry analysis of human SESN2 revealed three highly conserved phosphorylation sites. Importantly, mutation of these sites to phosphor-mimetic amino acids in exogenously expressed SESN2 promoted its interaction with GATOR2 and dramatically repressed mTORC1 [21]. This supports a potential relevance for SESN2 phosphorylation in the negative regulation of mTOR to prevent age-related pathologies caused by chronic mTOR activation.

## 3. SESN2 in Antioxidant Defence

**Figure 2:**
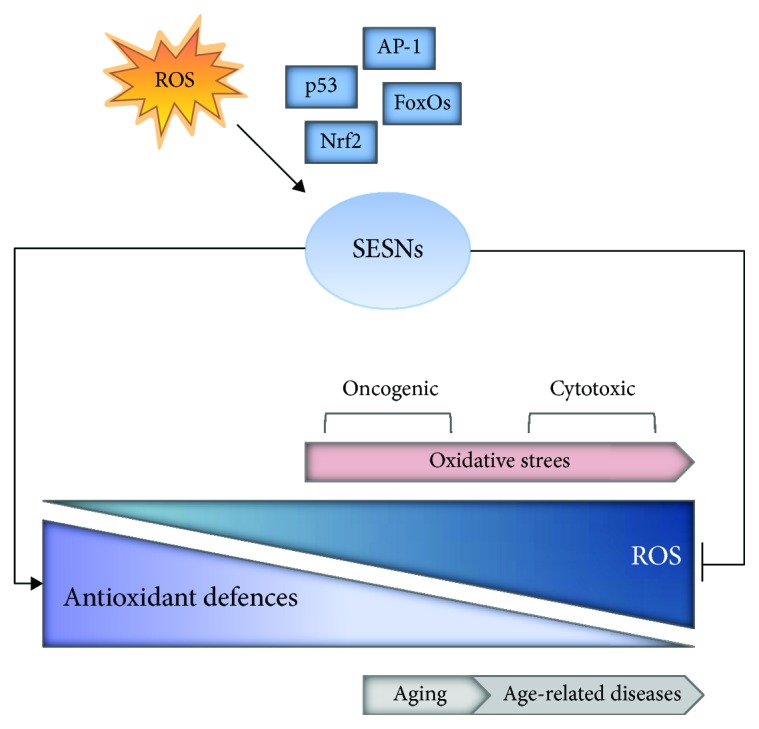
SESNs are master regulators of antioxidant defences. SESNs may be induced upon intracellular oxidative stress by the stimulation of transcription factors, such as p53, Nrf2, AP-1, and FoxOs. Once activated, SESNs limit ROS and oxidative stress by multiple mechanisms, thus having a functional role in age-related diseases as well as cancer.

SESNs promote antioxidant adaptive responses in cells as induced upon intracellular oxidative stress by the stimulation of transcription factors, such as p53, Nrf2, AP-1, and FoxOs (Figure 2). SESN1 is induced by hydrogen peroxide in a p53-dependent manner, whereas the induction of SESN2 by oxidative stress is only partially dependent on p53 activation [22]. SESN2 can be induced in conditions of increased ROS production, in a CCAAT/enhancer binding protein-beta- (C/EBP*β*-) dependent manner [23]. More recently, oxidative stress was found to induce SESN2 via activation of the transcription factor Nrf2 [17] and via the JNK/AP-1 signalling axis [18], while SESN3 is stimulated by oxidative damage via activation of FoxO transcription factors [24, 25]. Consistent with a relevant contribution of SESNs in the cellular antioxidant defence, silencing of all the three SESN isoforms blunts the antioxidant response to different oxidative stress challenges [16, 26], whereas ectopic expression of SESNs has a protective impact. However, the molecular underpinnings of this activity are still incompletely clear.

As mentioned above, the oxidoreductase active site within SESN-A is critical for the antioxidant function of hSESN2. While structural and biochemical evidence demonstrates that hSESN2 has intrinsic peroxidase activity, the physiological ROS substrate(s) of hSESN2 is (are) currently unknown. Hydrogen peroxide, which is among the most abundant and biologically significant cellular ROS species, is not efficiently reduced by hSESN2, and the only known substrate under *in vitro* conditions for hSESN2 is cumene hydroperoxide, which is not a physiological ROS in any known cell type [10].

Since a small conserved region in SESNs shows a limited sequence homology to AhpD protein of *Mycobacterium tuberculosis* [26], this similarity was analyzed for its ability to reduce known substrates of AhpD, such as peroxiredoxin (Prx). SESNs can indeed reduce Prx, but intriguingly this does not require their catalytic activity [27]. It was hypothesized that SESNs may promote the activity of other oxidoreductases, such as sulforedoxin (Srx), that in turn can regenerate Prx. Indeed, one recent study showed that SESNs can increase Srx expression through positive feedback activation of Nrf2, a transcription factor that orchestrates antioxidant responses [28].

Independent of their Prx-regulating activity, SESNs contribute to cell redox homeostasis through the regulation of AMPK-mTORC1 signalling pathways. dSESN^−/−^ Drosophila cells exhibit increased TOR activity [14]. Since mTOR activity may enhance ROS production via inhibition of autophagy or directly acting on mitochondrial function, a relevant share of the antioxidant role of SESNs may be derived from mTOR signalling inhibition.

## 4. SESNs as Regulators of Autophagy and Mitophagy

The term “autophagy” refers to a group of regulated mechanisms by which cells break down specific building blocks and structures, enabling their recycling, the rerouting of their energy, or their disposal when they are damaged and/or toxic [29]. While a number of pathway variants exist, the core machinery and the regulatory cascade its components map to are rather defined and evolutionarily conserved [29]. The Unc51-like kinases ULK1/2 are major phosphor-regulated initiator switches of the pathway, tightly controlled by positive growth signalling [30]. Their dephosphorylation triggers the nucleation of the phagophore, the precursory membrane structure from which the autophagic vesicle is formed, by a Vps14/Beclin1/Atg14L complex [31]. This event initiates a signalling cascade that accumulates PE-conjugated Atg8, which drives the maturation and closure of autophagosomes, enabling the docking of specific cargos and adaptor proteins, such as sequestosome-1/p62 [32]. The fusion of the autophagosome with the lysosomal compartment is mediated by multiple proteins, including SNAREs and UVRAG [33]. Autophagy is tightly linked to ROS control in the cell [34].

Mitophagy is a specialized form of autophagy by which dysfunctional or damaged mitochondria are selectively targeted by autophagosomes and delivered to lysosomes to be recycled by the cell. Hence, mitophagy represents an essential quality control mechanism to ensure the integrity and functionality of the mitochondrial network [35]. Dysregulation of mitophagy leads to unresolved damage to mitochondria, a powerful source for pathological ROS levels [36]. High levels of mitochondrial ROS lead in turn to an inflammatory status through various mechanisms, including hyperactivation of the NLRP3 inflammasome [36]. These events have been proposed to play a role in diverse degenerative pathologies by leading to the release of mitochondrial DNA, tissue injury, and cell death [37, 38]. Several reports indicate that SESNs are positive regulators of autophagy in the face of diverse environmental stresses that entail mitochondrial dysfunction [28, 39–41]. Under energetic stress, AMPK-dependent ULK1 phosphorylation promotes autophagy by targeting several downstream crucial autophagy effectors involved in autophagy machinery [42]. p62/SQSTM1 protein is another important player in the autophagy process. Indeed, p62 can function as an adaptor protein binding to diverse autophagy substrates, such as ubiquitinated proteins, damaged mitochondria, and the Nrf2 inhibitor Keap1, and promotes their autophagic degradation, contributing to the restoration of the physiological levels of ROS [43].

SESN2 binds to p62 and promotes the autophagic degradation of p62-dependent targets, including Keap1, thereby channelling autophagy turnover flux towards functionally coherent substrate sets and upregulating the transcription of antioxidant genes [28]. In addition, SESN2 physically associates with ULK1 and p62 to form a functional complex, favouring ULK1-mediated p62 phosphorylation [44]. Furthermore, SESN2 promotes the targeting of mitochondria for recognition by the autophagic machinery, a process described as “mitochondrial priming,” which is further potentiated by the intrinsic capability of SESN2 to increase ULK1 protein levels. This activity has been proposed to contribute to immunological homeostasis and the attenuation of NLRP3 hyperactivation [45]. Recently, the role of SESN2 in the protection of renal tubules during acute kidney injury (AKI) has been explored [46]. AKI is a pathophysiological condition characterized by increased mitochondrial damage that leads to oxidative stress and apoptosis in tubular renal cells [47]. Intriguingly, autophagy and mitophagy are induced in renal tubules in AKI through a p53-SESN2 axis, which may constitute a protective mechanism of the cell [46].

## 5. Control of the Cellular Energetic Metabolism by SESNs: Regulation of mTOR and AMPK Signalling

mTORC1 is one of the major signalling nodes coupling environmental and metabolic signals such as nutrients, growth factors, oxygen, and stress with the control of protein synthesis, lipid anabolism, and cell growth [48–50]. The recycling and scavenging of cell components prevent *de novo* a cell mass increase, and a major signal output from mTORC1 blunts autophagy [51]: mTORC1 phosphorylates ULK1 on Ser757, disrupting its interaction with AMPK, an event required to activate ULK1 to induce the canonical autophagic pathway [42].

A number of studies suggest that SESNs can serve as endogenous sensors of amino acid fluctuations in the cellular microenvironment, which are one of the central inputs regulating mTORC1 signalling. Indeed, SESN2 induction is necessary for cell survival during glutamine deprivation [52]. A SESN-dependent and AMPK-independent mechanism for mTORC1 inhibition might be mediated by the interaction of SESNs with GATOR2 [53–55]. As a result of this interaction, SESNs suppress lysosomal mTOR localization in a Rag-dependent manner. This mechanism is thus a potential integral component of mTORC1 regulation by amino acid availability, connecting stress responses with mTORC1 control [53–55]. SESNs also bind to the heterodimeric RagA/B-RagC/D GTPases, thus acting as GDIs for RagA/B [56]. Accordingly, ectopic overexpression of SESNs in HEK293T, HeLa, and MEF cell lines can inhibit amino acid-induced translocation of Rag-GEF and mTORC1 to the lysosome [56].

Intriguingly, two studies contribute biochemical and biophysical evidence suggesting that SESN2 acts as a direct sensor of leucine, which directly precludes the interaction of SESN2 with GATOR2 [11, 13, 57]. This intriguing hypothesis however has not been fully proven, and other studies question the idea that SESN2-dependent suppression of mTORC1 signalling is sensitive to leucine [14, 24, 53, 54, 56, 58–61], and the physical interaction between SESN2 and GATOR2 in cells can still be detected despite leucine-rich culture conditions [53–55]. A possible scenario to reconcile these conflicting observations might rely on the effect of leucine on SESN2 being highly context-dependent. Further studies will clarify the impact of leucine levels on the SESN2-GATOR2 interaction across conditions. Additional mechanisms have been proposed to explain the contribution of SESN2 in amino acid sensing. Ye et al. reported a signalling axis whereby the eIF2*α* kinase General Control Nonderepressible 2 (GCN2) is the driving sensor of amino acid scarcity and translationally upregulates the stress transcriptional regulator ATF4 to induce the expression of SESN2, which in turn may block the lysosomal recruitment and activation of mTORC1 [62]. Another report shows that in response to leucine starvation, SESN2 is phosphorylated by ULK1 thus affecting mTORC1 activation [21]. In addition to being phosphorylated by ULK1, SESN2 may sustain ULK1-dependent phosphorylation [44] and degradation of SQSTM1/p62 [28]. Because SQSTM1 is an mTORC1 regulator involved in amino acid sensing [63–65], deeper studies will be needed to clarify the functional link between SESN2, ULK1, SQSTM1, and GATOR2 in the context of amino acid sensing.

Notably, amino acid consumption (particularly that of amino acids entering anaplerotic routes as alternative intermediates for anabolism) and derived imbalances can constitute sources of redox alteration. For example, Byun et al. found that glutamine deprivation reduces GSH synthesis (possibly because of a subsequent shortage in 2-oxoglutarate), which in turn induces SESN2 expression via the ROS-p38 MAPK-C/EBP pathway. Of note, this study identified a positive feedback loop between SESN2 and mTORC2 as necessary to suppress mTORC1 activity in glutamine-depleted non-small-cell lung cancer cells (NSCLC). This divergent regulation of mTORC1 and mTORC2 by SESN2 might contribute to the maintenance of redox homeostasis in the face of amino acid imbalance, enabling lung cancer cells to survive under glutamine-depleted conditions, and might thus constitute an attractive synergistic therapeutic target in tumours showing such metabolic “addiction” [66].

AMPK inhibits mTORC1 through the phosphorylation of TSC2 and raptor (two phosphor-dependent hallmarks of mTORC1 inactivation) in response to cellular energy cues [67–69]. Evidence indicates that SESN-dependent AMPK induction is important for mTORC1 suppression in diverse cellular contexts, and at least one SESN paralog, SESN2, has been found to coimmunoprecipitate with AMPK from tissue extracts [40, 59, 70–73]. In *Drosophila*, chronic TOR activation upregulates dSESN through accumulation of ROS. dSESN acts then as a negative feedback regulator of TOR function. In accordance, mammalian SESNs sustain the AMPK-TSC2 axis that integrates metabolic and stress inputs and prevents age-associated pathologies caused by chronic TOR activation [14]. SESN1 and SESN2 are negative regulators of mTOR signalling through the activation of AMPK and TSC2 phosphorylation in a p53-dependent manner, and this negative regulation is required for mTORC1 signalling suppression upon DNA damage [58]. In response to genotoxic stress, SESN2 expression is upregulated favouring sustained AMPK activity by orchestrating the recruitment of LKB1, as well as increasing LKB1/AMPK*αβγ* expression. In addition, both AMPK and SESN2 coordinate to suppress Akt-mTOR signalling as induced by ionizing radiation, thus acting as radiation sensitizers in MCF7 breast cancer cells [60]. The SESN2-dependent induction of AMPK activity and blunting of mTORC1 seem to bear substantial potential relevance to attenuate cell damage in conditions associated with acute ischemia, such as hypoxic-ischemic encephalopathy (HIE, caused by decreased oxygen and cerebral blood flow to the brain) or cardiomyocyte ischemia [74]. Exogenous elevation of SESN2 levels reduced the brain infarct area and brain atrophy and improved long-term neurological function in a neonatal rat model of HIE through the induction of AMPK signalling and subsequent blockade of mTOR signalling [75]. SESN2 also exerts neuroprotective effects during cerebral ischemia/reperfusion injury, a complex pathophysiological process characterized by enhanced levels of ROS and apoptosis, possibly by increasing mitochondrial biogenesis through an AMPK/PGC-1*α* pathway, which attenuates oxidative stress [76, 77]. SESN2 also accumulates in the heart during ischemic conditions, stabilizing an LKB1-SESN2-AMPK complex that cannot be assembled in SESN2-KO hearts, blunting AMPK during ischemic injury [70]. Somewhat expected by the prominent role that AMPK signalling has in counteracting high-glucose-induced damage and insulin resistance, SESN2 might contribute to the amelioration of disorders derived from metabolic syndrome and hyperglycaemia, such as hyperglycaemia-induced glomerular injury [73, 78], hepatosteatosis, and insulin resistance [79]. Of note, SESN2 is induced upon hypernutrition, and the genetic ablation of SESN2 exacerbates obesity-induced mTORC1-S6K activation, glucose intolerance, insulin resistance, and hepatosteatosis in the liver of obese mice, all of which are rescued by AMPK pharmacological activation [80].

SESN-driven regulation of AMPK and mTOR signalling also bears relevance for tumour biology. SESN2 and SESN3 suppress NK-92 cell-mediated cytotoxic activity on ovarian cancer cells through the induction of AMPK and inhibition of mTORC1, pointing at its potential relevance for immunotherapy in ovarian cancer [81]. SESN2 is decreased in colorectal cancer (CRC) [82], and its overexpression limits ROS production, inhibits cell growth, and stimulates apoptosis in CRC cell lines [83]. In addition, a protective role of SESN2 in gentamicin-induced stress has been reported. Genetic ablation of SESN2 enhances the sensitivity of hair cells to gentamicin suggesting that it may be involved in protecting sensory hair cells against gentamicin via modulation of the AMPK/mTOR pathway [84]. Further supporting a functional link between SESNs and AMPK signalling in cancer cells, GOF mutant p53 blocks a SESN1/AMPK/PGC-1*α*/UCP2 axis, increasing mitochondrial superoxide production in cancer cells [85].

**Figure 3:**
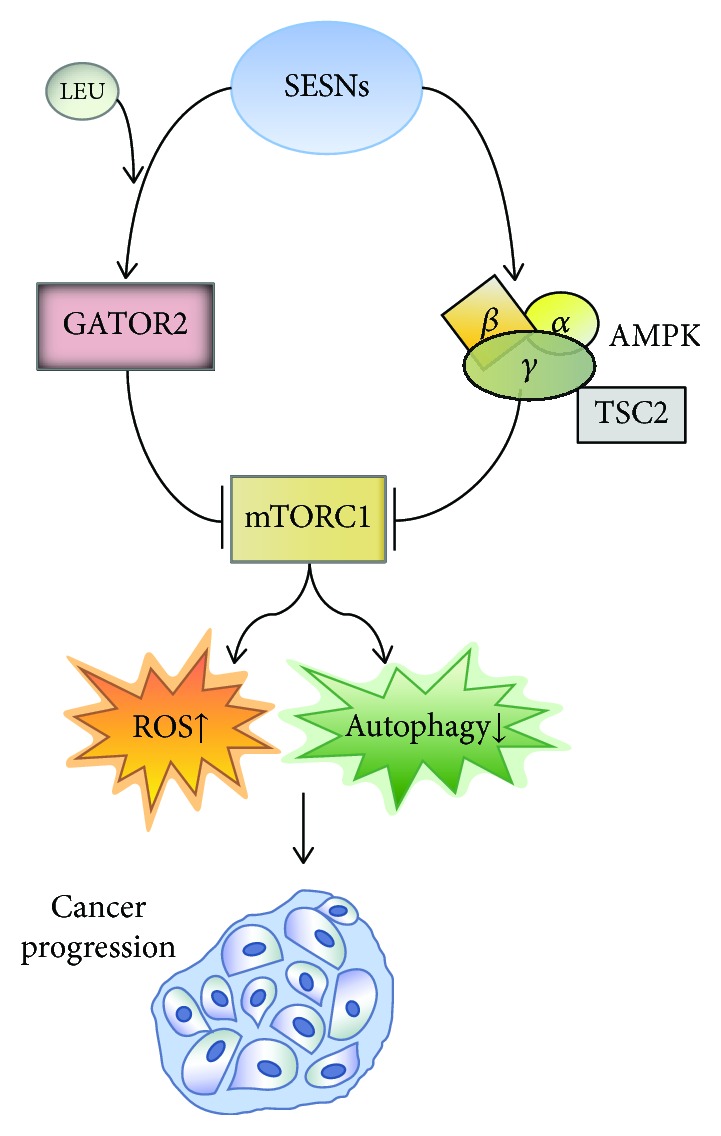
mTORC1 regulation by SESNs and its role in cancer progression. SESNs act as a master regulator of mTORC1 function through activation of GATOR2 (in the presence of leucine) and AMPK activation, thus playing a tumour-suppressive role.

In summary, SESNs might play an important role in signalling networks linking nutrient starvation to the control of major growth signalling and energy management networks (i.e., mTORC1 and AMPK) (Figure 3). While a substantial body of evidence has been gathered during the study of tumour biology, these concepts highlight the pivotal relevance of SESN-driven networks in the unfolding of processes related to aging.

## 6. SESNs in Aging

Dysregulation of mTORC1 and deactivation of AMPK signalling [86, 87], associated with increased ROS production or decreased ROS surveillance, are all closely related to hallmarks of aging, such as loss of metabolic homeostasis and proteostasis and reduced muscle function [88, 89]. Moreover, since SESNs are key regulators of several of these processes, they are also involved in aging. In *Caenorhabditis elegans*, SESN1 gene mutants show ROS accumulation, muscular cell abnormalities, and reduced lifespan [90]. At the same time, gain-of-function mutants induced increased lifespan with decreased muscle ROS. dSESN^−/−^ flies exhibit chronic suppression of AMPK and activation of mTORC1, resulting in fat accumulation, blood sugar elevation, and skeletal and cardiac muscle degeneration [14]. In mice, however, SESN2 loss-of-function mutants have normal aging, perhaps reflecting different and multifactorial mechanisms of aging in invertebrates with respect to mammals [6]. Nonetheless, SESN level is modulated by aging. Older men show less SESN1 and SESN3 protein level in skeletal muscle and reduced amount of the phosphorylated *δ*-isoform of SESN2 compared to middle-aged and young men [15]. In the same experimental conditions, the mRNA expression of SESNs was highly upregulated, probably for compensatory mechanisms.

## 7. SESNs and Toxic Stress Tolerance

Protection from the damage exerted by xenobiotics is paramount for organismal homeostasis. SESNs may have an important role in counteracting toxic chemicals through the regulation of oxidative stress and autophagy induction [14, 26].  Table 1 summarizes relevant studies reporting a role for SESNs in neuronal stress tolerance after toxic injuries. Notably, SESN2 is expressed in the central nervous system, and evidence supports that it is an important contributor to antioxidant systems in the brain [23]. For example, dysregulation of SESN expression in different conditions, such as neurodegenerative syndromes associated with advanced HIV infection, causes increased oxidative stress [91]. Moreover, SESN protein levels were found to be higher in patients suffering from Alzheimer's and Parkinson's diseases [92, 93], further highlighting their role as components of oxidative stress signatures and homeostatic surveillance in brain tissue. The heavy metal chromium (Cr) exists in different oxidation states and causes harmful effects on the organism. Reduction of Cr(VI) to Cr(III) leads to various reactive Cr intermediates and free radical generation, which results in oxidative stress [94]. Cr(VI) was reported to inhibit acetylcholinesterase activity in rats leading to oxidative damage to their brain [95]. In *Drosophila melanogaster*, Cr(VI) also causes neuronal cell death via ROS generation [96]. A recent study reported that oxidative stress, apoptosis, and neuronal cell death observed upon exposure of *D. melanogaster* larvae to Cr(VI) were reverted by ectopic SESN overexpression. Moreover, SESN overexpression enhances autophagy flux and decreases mTORC1 signalling/p-S6k levels in neuronal cells, suggesting an increased catabolic activity and cellular repair [97].

1-Methyl-4-phenylpyridinium (MPP+) is a metabolite derived from 1-methyl-4-phenyl-1,2,3,6-tetrahydropyridine (MPTP) that selectively destroys dopaminergic neurons in the substantia nigra of the brain by inducing mitochondrial dysfunction, oxidative stress, and apoptosis [98]. Because of its powerful neurotoxic activity, it is often used as a tool for studying Parkinson's disease (PD) in various animal models [99]. Of note, MMP+ induces the expression of SESN2 in SH-SY5Y human neuroblastoma cells and this induction is mediated by p53 [92]. Furthermore, knockdown of SESN2 enhances MPP+-induced neurotoxicity by triggering oxidative stress, mitochondrial dysfunction, apoptosis, and cell death [92]. Thus, the induction of SESN2 exerts a protective effect in neurons that might be effective for the therapeutical management of PD and other neurodegenerative diseases.

Sevoflurane, one of the most commonly used volatile anaesthetics in clinical treatment, can induce neuronal degeneration and cognitive impairment through ER stress-driven apoptosis in neurons of aging rats [100]. Notably, sevoflurane treatment induces SESN2 expression in a p53-dependent fashion in human neuroblastoma M17 cells, and knockdown of SESN2 blunts superoxide activity, unleashing oxidative stress and apoptosis [88].

Accumulation of amyloid *β*-peptide (A*β*) in senile plaques represents a pathological hallmark of Alzheimer's disease and leads to neurodegeneration by inducing cytotoxicity and oxidative stress [101]. A recent study explored a potential link between SESN2 and A*β* neurotoxicity. Chen et al. reported that SESN2 expression and the autophagy marker LC3B-II were elevated in primary rat cortical neurons upon A*β* exposure. Importantly, downregulation of SESN2 by siRNA abolished LC3B-II formation caused by A*β* and was accompanied by neuronal death. Accordingly, inhibition of autophagy by bafilomycin A1 also enhanced A*β* neurotoxicity [41]. This indicates that SESN2 induced by A*β* plays a protective role against A*β* neurotoxicity through the positive regulation of autophagy.

SESN2 also features potential beneficial activities on both vascular endothelial and liver injuries, highlighting its potential as a therapeutical target in cardiovascular and liver diseases [102, 103]. Activation of angiotensin II (AngII) signalling can trigger endothelial cell dysfunction, including proinflammatory adhesion molecules, production of ROS, and endothelial apoptosis. Again, supporting its relevant role as a component of stress adaption signatures, SESN2 expression is increased by AngII and knockdown of SESN2 enhances AngII-induced toxicity in human umbilical vein endothelial cells (HUVECs) [102].

Acetaminophen (APAP) is one of the most commonly used analgesic/antipyretic drugs, and at high doses, it can provoke acute hepatotoxicity, resulting in serious liver damage and death [104]. SESN2 exerted a protective effect against APAP-induced acute liver damage by inhibiting oxidative stress and proinflammatory signalling, possibly through the inhibition of downstream MAPK pathway activation [103]. It will be interesting in the future to explore the contribution of other pathways downstream of SESN2 that have also been closely related mechanistically to APAP toxicity, such as mitophagy [105].

## 8. Conclusions

In recent years, remarkable progress has been made towards understanding the biochemical mechanisms behind the role of SESNs in physiopathology. Sestrins are integrated as components of adaptive responses against a variety of cellular stresses, including DNA damage, oxidative stress, hypoxia, and metabolic stresses. Thus, SESNs are now recognized as key regulators of cellular metabolism and indispensable contributors to cellular homeostasis in normal physiology and diseased states. As a positive regulator of AMPK and a repressor of mTORC1, SESNs elicit protective effects on various metabolic disorders such as diabetes and obesity, cancer, cardiac hypertrophy, and atherosclerosis.

Importantly, modulation of SESNs can activate an autophagic response, which in turn may lead to cancer cell death, as well as a cytoprotective effect in a manner highly dependent by the metabolic and environmental context in which tumour cells reside. Moreover, the potent antioxidant and autophagic effects orchestrated by SESNs confer neuronal stress tolerance to toxic injuries and neuroprotection in neurodegenerative disorders that are closely linked to oxidative stress, such as Parkinson's disease and Alzheimer's disease. Future studies are required to further validate such potential of SESNs as biomarkers and therapeutic targets against these disorders and to fully understand how their multiple functions are interrelated and coordinated at system level.

## Figures and Tables

**Table 1:** Some examples of the cytoprotective effect by SESNs.

Entry	Drugs or toxic substances	Cell lines	Tissue type/model organism	Effect on SESNs	Molecular mechanisms	Biological effect	Refs
1	Amyloid *β*-peptide	Primary rat cortical neurons	Transgenic mice	SESN2 ↑	LC3B-II ↑	Protective autophagy, prevention of neuronal cell death, protection against Alzheimer's disease	[41]
2	Sevoflurane	M17	Neuroblastoma	SESN2 ↑	p53-dependent mechanism	Prevention of neuroapoptosis and ROS	[88]
3	1-Methyl-4-phenylpyridinium	SH-SY5Y	Neuroblastoma	SESN2 ↑	p53-dependent mechanism	Protection against oxidative stress, mitochondrial dysfunction, apoptosis, and cell death and protection against Parkinson's disease	[92]
4	Chromium IV	Neuronal cells	Drosophila melanogaster larvae	dSESN ↑	ATG-8 ↑, p-JNK, p-Akt, p-FoxO, cleaved caspase-3 ↓, TOR/p-S6k ↓	Protection against oxidative stress, apoptosis, and neuronal cell death and protective autophagy	[97]
5	Angiotensin II	HUVECs	Endothelial	SESN2 ↑	JNK/c-Jun pathway ↑	Protection against cardiotoxicity of angiotensin II	[102]
6	Acetaminophen	Liver cell	Mice	SESN2 ↑	JNK, ERK pathway ↓, p38 ↓	Inhibition of oxidative stress, proinflammatory signalling, protection against liver toxicity	[103]
